# Physician Variability in Time to Kidney Biopsy and Glucocorticoid Prescription in IgA Nephropathy: A Population-Based Cohort Study

**DOI:** 10.34067/KID.0000001232

**Published:** 2026-05-14

**Authors:** Bryce Barr, Hailey Hildebrand, Ian W. Gibson, Navdeep Tangri

**Affiliations:** 1 Department of Medicine, Max Rady College of Medicine, University of Manitoba, Winnipeg, Manitoba, Canada; 2 Chronic Disease Innovation Centre, Seven Oaks General Hospital, Winnipeg, Manitoba, Canada; 3 Department of Pathology, Max Rady College of Medicine, University of Manitoba, Winnipeg, Manitoba, Canada

**Keywords:** epidemiology and outcomes, GN, IgA nephropathy

## Abstract

**Key Points:**

- Similar patients experienced almost three-fold differences in biopsy timing depending on which physician they saw.
- Glucocorticoid prescribing varied meaningfully by physician after adjustment for clinical and biopsy features.

**Background:**

Practice patterns in the management of IgA nephropathy (IgAN) vary but the extent of physician-level variability in key decisions, such as kidney biopsy and glucocorticoid use, is unknown. Understanding variability in clinical practice provides essential context for knowledge translation as new evidence and guidelines emerge for the treatment of IgAN. We hypothesized that time to biopsy and likelihood of glucocorticoid use would vary significantly by physician.

**Methods:**

We conducted a population-based cohort study of adults with biopsy-proven IgAN in Manitoba, Canada (2002–2021). Linear mixed-effects models were used to examine factors associated with time from urinary abnormality to kidney biopsy, and logistic mixed models were used to assess the likelihood of glucocorticoid prescription. Models included random intercepts for physician to quantify between-physician variability using the intraclass correlation coefficient (ICC), median time ratio, and median odds ratio.

**Results:**

Among 380 patients managed by 38 physicians, the physician-level ICC for time to biopsy was 0.31, indicating that nearly one third of the total variability in time to biopsy was attributable to between-physician differences. The corresponding median time ratio was 2.65, representing a nearly three-fold difference in time to biopsy between two otherwise similar patients managed by different physicians. For glucocorticoid prescription, the ICC was 0.07, indicating 7% of the variability was attributable to physician practice. The median odds ratio was 1.59, indicating that two otherwise similar patients could have 59% different odds of being prescribed glucocorticoids depending on which physician managed their care.

**Conclusions:**

This is one of the first studies to quantify between-physician variability in IgAN care. Substantial between-physician variability exists in the timing of biopsy and use of immunosuppression in IgAN. These findings highlight opportunities for targeted knowledge translation to promote timely, equitable, and evidence-aligned care.

## Visual Abstract

**Figure:**
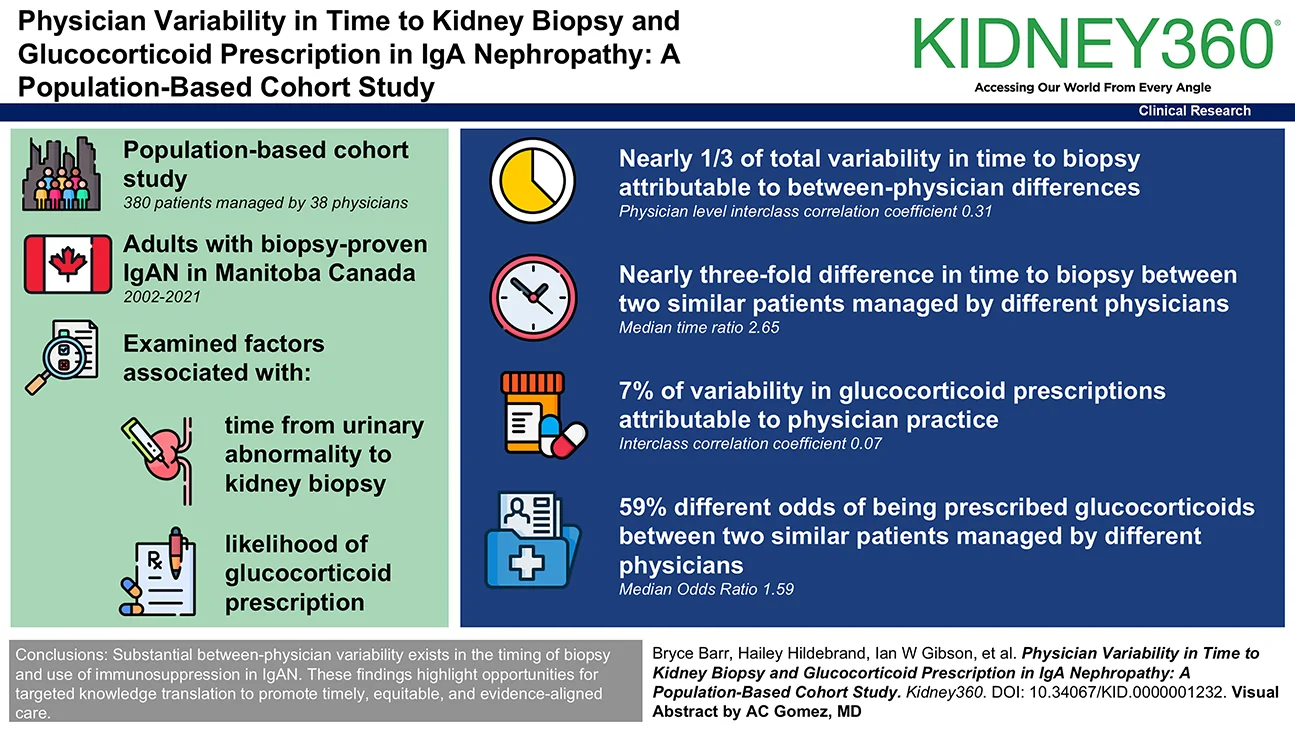

## Introduction

IgA nephropathy (IgAN) is a leading cause of CKD and kidney failure worldwide.^1,2^ At the time of the 2021 Kidney Disease Improving Global Outcomes (KDIGO) Clinical Practice Guideline for the Management of Glomerular Diseases, patients with <1 g/d of proteinuria were considered to have a favorable long-term prognosis,^3^ and treatment was centered on renin-angiotensin system inhibitors, with immunosuppressive therapy reserved for persistent proteinuria despite optimized supportive care.^4–8^

Since 2021, epidemiologic studies with long-term follow-up have shown that the risk of kidney failure in IgAN remains elevated at lower levels of proteinuria than previously recognized,^9–11^ while randomized trials have demonstrated that glucocorticoids and other immunosuppressive therapies reduce the risk of kidney function decline,^12–15^ with additional agents in development.^16^ Reflecting this growing evidence base, the KDIGO 2025 Clinical Practice Guideline for the Management of IgA Nephropathy lowered the proteinuria threshold at which patients are considered at risk and places greater emphasis on immunosuppressive therapy within treatment algorithms.^17^

In this context, understanding preexisting physician practice patterns is critical for interpreting the uptake of emerging evidence and increasing implementation of updated guidelines. Accordingly, we aimed to characterize physician practice patterns in a population-based cohort of patients with IgAN from 2002 to 2021, focusing on the timing of kidney biopsy, glucocorticoid prescribing, and between-physician variability in these key decisions.

## Methods

### Objectives

The first research question was as follows: Among adult patients with biopsy-proven IgAN in Manitoba, what are the factors associated with time to kidney biopsy, and how much does time to kidney biopsy vary by physician?

The second research question was as follows: Among adult patients with biopsy-proven IgAN in Manitoba, what are the factors associated with the likelihood of receiving glucocorticoids, and how much does this vary by physician?

### Study Design and Data Sources

This was a retrospective cohort study arising from the Manitoba Glomerular Diseases Registry.^18^ Briefly, the registry identifies all patients who have undergone a kidney biopsy in the Canadian province of Manitoba since January 2002; their complete pathology data are then linked to the data repository at the Manitoba Centre for Health Policy (MCHP).^19^ The variables captured and data sources in the registry are summarized in Supplemental Tables 1 and 2. Patients older than 18 years biopsied between January 1, 2002, and December 31, 2021, were considered for inclusion in this study. Patients who could not be linked to administrative data, those on dialysis at the time of their initial consultation or kidney biopsy, or who underwent a kidney biopsy before nephrology involvement were excluded. For the analysis of time to kidney biopsy, those whose initial nephrology visit was an inpatient consultation were excluded.

For the first research question, the index date was the date of initial nephrology consultation, identified using physician billing codes (Supplemental Table 3). For the second research question, the index date was the date of the kidney biopsy, which is identified directly in the registry pathology data.

### Covariates

Baseline eGFR was calculated from the most proximal serum creatinine measurement within 90 days of the index date, using the CKD Epidemiology Collaboration 2021 equation.^20^ Baseline proteinuria was the most proximal 24-hour urine protein measurement within 90 days of the index date; when only spot urine protein–creatinine ratio was available, values were converted to a 24-hour measurement using the method described by Hogan *et al.*^21^ When only urine albumin–creatinine ratio was available, the value was converted to PCR using a factor of 1.37,^22,23^ followed by a conversion to the 24-hour value. In this administrative dataset, hematuria is not accurately captured and therefore could not be included as a covariate. Location of residence and socioeconomic status were derived from dissemination area-level information generated from the Canadian Census.^24^ The presence of hypertension and diabetes were obtained from Canadian Chronic Disease Surveillance System definitions^25^ (Supplemental Table 3). Era of diagnosis was prespecified according to the publication date of two landmark randomized trials of glucocorticoids in IgAN because it was hypothesized that these trials may have resulted in a change in physician behavior. Patients diagnosed before the publication of the Manno trial^26^ (2009 and previous) were classified as era 1. Those diagnosed between 2010 and 2015 (inclusive), after Manno but before the Supportive Versus Immunosuppressive Therapy for the Treatment Of Progressive IgA Nephropathy (STOP-IgAN) trial,^27^ were classified as era 2. Patients diagnosed from 2016 to 2021, after Supportive Versus Immunosuppressive Therapy for the Treatment Of Progressive IgA Nephropathy, were classified as era 3. Finally, the Oxford classification was not reported in our center before 2016. As the registry captures the number of crescents, number of glomeruli, and amount of interstitial fibrosis and tubular atrophy (IFTA), covariates were created to approximate the Oxford T and C scores. Very few patients had >25% crescents, and therefore C1 and C2 were combined for analysis. Physician identity was determined using a unique billing identifier and was anonymized before analysis.

### Outcomes

For research question 1, the primary outcome was time, in days, from the first nephrology visit to kidney biopsy. Time to biopsy was right skewed and was thus log transformed.

For research question 2, the primary outcome was a minimum 28-day prescription for glucocorticoids (identified by anatomical therapeutic chemical code H02) within a prespecified window after the kidney biopsy (0–365 days); only prescriptions with a mean daily dose over the 28-day period of >20 mg per day were considered eligible, to minimize misclassification of low-dose or short-course glucocorticoid use prescribed for non-IgAN indications. Glucocorticoid exposure was modeled as a binary outcome rather than time to prescription because the timing of initiation may be influenced by postbiopsy factors and patients treated soon after biopsy may be systematically different from those treated later.

### Statistical Analyses

Continuous variables were reported as median and interquartile range (IQR). Categorical variables were reported as percentages. We modeled the association of clinical factors and time between nephrology visit and kidney biopsy using mixed-effects multivariable linear regression. Fixed effects included age, comorbidities, rurality, and baseline eGFR and proteinuria. A random intercept for physician was specified to account for clustering of patients by treating nephrologist. Five models were fit to assess the change in between-physician variability after adjusting for the effect of covariates. We attempted to include the preconsultation eGFR and proteinuria values in the models and express them as preconsultation slopes conditional on the baseline values (to limit collinearity with the baseline values). However, approximately half the patients did not have sufficient laboratory data to calculate such slopes, so these parameters were not included as covariates. For interpretability, coefficients from the log-linear model were exponentiated and presented as time ratios and corresponding percent differences.

Between-physician variability was expressed using intraclass correlation coefficient (ICC),^28^ which quantifies the proportion of the variability in time to kidney biopsy attributable to between-physician differences. In addition, we derived the median time ratio (MTR) as an interpretable analog to the median odds ratio (MOR) commonly used in multilevel logistic regression.^29^ The methods for calculating the ICC and MTR are demonstrated in the Supplemental Methods. Confidence intervals (CIs) for the ICC and MTR were estimated using parametric bootstrap with 1000 replications.

We modeled the association of covariates with the likelihood of glucocorticoid prescribing within the first year after biopsy using a mixed-effects multivariable logistic regression model. Fixed effects included age at biopsy, sex, baseline proteinuria, baseline eGFR, diabetes, hypertension, era of diagnosis, and Oxford C and T scores. A random intercept for physician was included to account for clustering of patients within physicians. Three models were built to assess the change in between-physician variability after adjusting for the effects of covariates.

We quantified physician variability in prescribing using the ICC and MOR,^29^ which expresses the median multiplicative increase in the odds of glucocorticoid prescribing between a higher prescribing physician versus lower prescribing physician, across all randomly sampled pairs of physicians. This value can be interpreted as the median increase in the odds of receiving glucocorticoids if an identical patient were treated by a higher versus lower prescribing physician. An MOR of 1 would indicate no variability in glucocorticoid prescribing between physicians. The method for calculating the MOR is demonstrated in the Supplemental Methods. CIs for the ICC and MOR were estimated using parametric bootstrap with 1000 replications.

To assess concordance with pre-existing KDIGO recommendations, we constructed a subset of patients meeting a proxy definition of steroid eligibility, which comprised patients with >1 g/d of proteinuria despite at least 90 days of treatment with renin-angiotensin system inhibitors. For this analysis, the outcome was dispensing of glucocorticoids within 365 days of the eligibility index date. A mixed-effects logistic regression with the same fixed effects and random intercept for physician was prespecified. However, only 66 patients met these criteria, and of them, only three received steroids. Therefore, the model could not be meaningfully fit. Additional details on this attempted analysis are provided in the Supplemental Methods.

We performed sensitivity analyses restricting to physicians with at least five patients in the cohort. The same model structures for both research questions were applied, and the effect estimates were compared with the full analysis set.

For the linear models, models were compared using the Akaike information criterion (AIC), as well as conditional R^2^ and likelihood ratio test. Model diagnostics included examination of residual plots, Q-Q plots, and distribution of residuals to validate model assumptions. For the logistic models, models were compared using AIC and likelihood ratio testing. Random effects were evaluated by examining the best linear unbiased predictors and distribution of random effects. Caterpillar plots were used to plot the random effects.

Missing data were assessed as missing at random, limited to baseline eGFR and proteinuria (<10% each), and were handled using multiple imputation with chained equations (five datasets). All analyses were conducted in R version 2025.05.1, with models built using the packages *nlme* and *lme4*, and permission from MCHP was obtained to use the results of the analysis for this study (Table 1).

**Table 1:** Characteristics of patients with IgA nephropathy at the time of biopsy by glucocorticoid treatment

Characteristic	Overall (*n*=380)	Untreated (*n*=291)	Treated (*n*=89)
Age at biopsy	42 (30–54)	42 (30–52)	43 (33–59)
Female sex	156 (41%)	119 (42%)	37 (42%)
eGFR at initial visit, ml/min per 1.73 m^2^	52 (29–82)	52 (31–83)	46 (21–65)
eGFR at biopsy, ml/min per 1.73 m^2^	47 (25–78)	47 (25–81)	42 (20–66)
Proteinuria at initial visit, g/d	1.88 (1.21–3.31)	1.79 (1.11–2.82)	2.88 (1.56–4.10)
Proteinuria at biopsy, g/d	1.71 (1.05–2.71)	1.60 (1.00–2.38)	2.41 (1.53–4.06)
Days to kidney biopsy, median (IQR)	63 (20–321)	85 (22–368)	34 (8–182)
Days to kidney biopsy, geometric mean (95% CI)	113 (93 to 137)	122 (99 to 151)	84 (53 to 134)
Diabetes	64 (17%)	45 (15%)	19 (21%)
Hypertension	210 (55%)	160 (55%)	50 (56%)
Rural dwelling	148 (39%)	114 (39%)	34 (38%)
**IFTA**			
0%–25%	175 (46%)	132 (45%)	43 (48%)
26%–50%	90 (24%)	66 (23%)	24 (27%)
>50%	115 (30%)	93 (32%)	22 (25%)
Crescents present	100 (26%)	52 (18%)	48 (54%)

Continuous variables are expressed as median (interquartile range) unless otherwise indicated.

Categorical variables are expressed as percentages. CI, confidence interval; IFTA, interstitial fibrosis and tubular atrophy; IQR, interquartile range.

## Results

**Figure 1:**
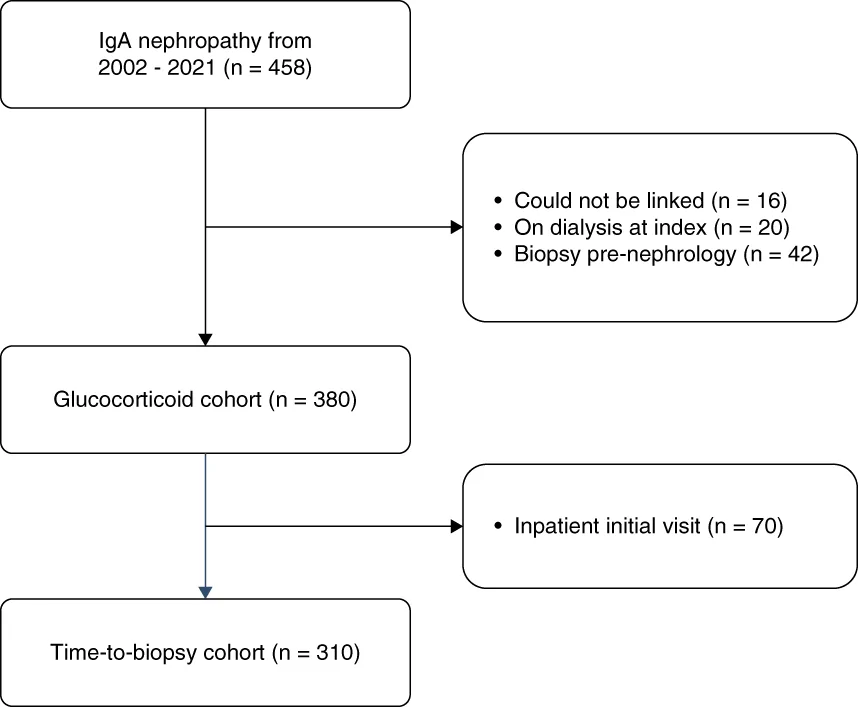
**Cohort flow diagram.** Among 458 patients with biopsy-proven IgAN between 2002 and 2021, 16 could not be linked, 20 were receiving dialysis at index, and 42 underwent biopsy before nephrology assessment, leaving 380 patients in the glucocorticoid cohort. For analyses of time to biopsy, a further 70 patients with an inpatient initial visit were excluded, yielding a final time-to-biopsy cohort of 310 patients. IgAN, IgA nephropathy.

A total of 458 patients with IgAN were identified from the registry. Of those, 14 could not be linked to administrative data, 20 were on dialysis at the time of their initial consultation or kidney biopsy, and 42 had a kidney biopsy ordered by another physician before their initial nephrology consult, leaving a total of 380 patients in the cohort (Figure 1), managed by 38 physicians, with a median of 7.5 patients per physician (IQR, 3–14). The median age at diagnosis was 42 (IQR, 30–54), and most were male (*n*=224, 59%). The median eGFR at kidney biopsy was 47 ml/min per 1.73 m^2^ (IQR, 25–78), while median proteinuria was 1.71 g/d (IQR, 1.05–2.71).

### Factors Associated with and Variability in Time to Biopsy

The initial nephrology visit was an inpatient consult for 70 patients; thus, 310 patients were included in the time-to-biopsy analysis. The characteristics of the patients in the time-to-biopsy cohort are summarized by era of diagnosis in Table 2. The characteristics of the patients who were seen as an inpatient versus an outpatient are presented in Supplemental Table 4.

**Table 2:** Characteristics of the time-to-biopsy cohort at the first nephrology visit

Characteristic	2002–2009 (*n*=71)	2010–2015 (*n*=71)	2016–2021 (*n*=168)
Age	41 (28–50)	42 (30–59)	43 (34–54)
Female sex	24 (34%)	32 (45%)	75 (45%)
eGFR at initial visit, ml/min per 1.73 m^2^	69 (41–91)	68 (35–93)	55 (33–88)
Proteinuria at initial visit, g/d	1.57 (0.92–2.69)	1.84 (0.97–3.04)	1.42 (0.80–2.49)
Rural dwelling	25 (35%)	32 (45%)	57 (34%)
**SES quintile**			
1	20 (28%)	26 (37%)	55 (33%)
2	19 (27%)	13 (18%)	32 (19%)
3	11 (15%)	13 (18%)	31 (19%)
4	13 (18%)	14 (20%)	30 (18%)
5	8 (11%)	5 (7.0%)	18 (11%)
Diabetes	7 (10%)	17 (24%)	39 (23%)
Hypertension	54 (76%)	64 (90%)	149 (89%)
Days to kidney biopsy, median (IQR)	96 (36–402)	98 (38–331)	75 (22–378)
Days to kidney biopsy, mean (SD)	366 (638)	464 (860)	635 (1395)

Continuous variables are expressed as median (interquartile range).

Categorical variables are expressed as percentages. IQR, interquartile range; SES, socioeconomic status.

Time to kidney biopsy was variable, with median time to biopsy 63 days (IQR, 20–321). The average (mean) time to biopsy increased from 366 days in era 1 (SD, 638) to 635 days in era 3 (SD, 1395). At the time of biopsy, 64 patients (17%) had diabetes, while 210 (55%) had hypertension. A total of 148 (39%) were rural dwelling at diagnosis. IFTA was prevalent; 90 patients (24%) had 26%–50% IFTA, equivalent to Oxford T1, while 115 (30%) had >50% IFTA, equivalent to Oxford T2. Cellular or fibrocellular crescents were present in 100 patients (26%).

We fit five multivariable mixed-effects linear regression models to assess the association of covariates with the time to kidney biopsy. Of these, the model with age, sex, eGFR, proteinuria, location of residence, and presence of diabetes and hypertension, along with an interaction term between proteinuria and location of residence, had the lowest AIC and highest marginal and conditional R^2^. Therefore, this model was considered the final model. As time to kidney biopsy increased across eras, era of diagnosis was evaluated as a candidate covariate in the final model; however, its inclusion did not meaningfully alter effect estimates or improve model fit and was therefore not retained in the final model. The candidate models are summarized in Supplemental Table 5.

**Figure 2:**
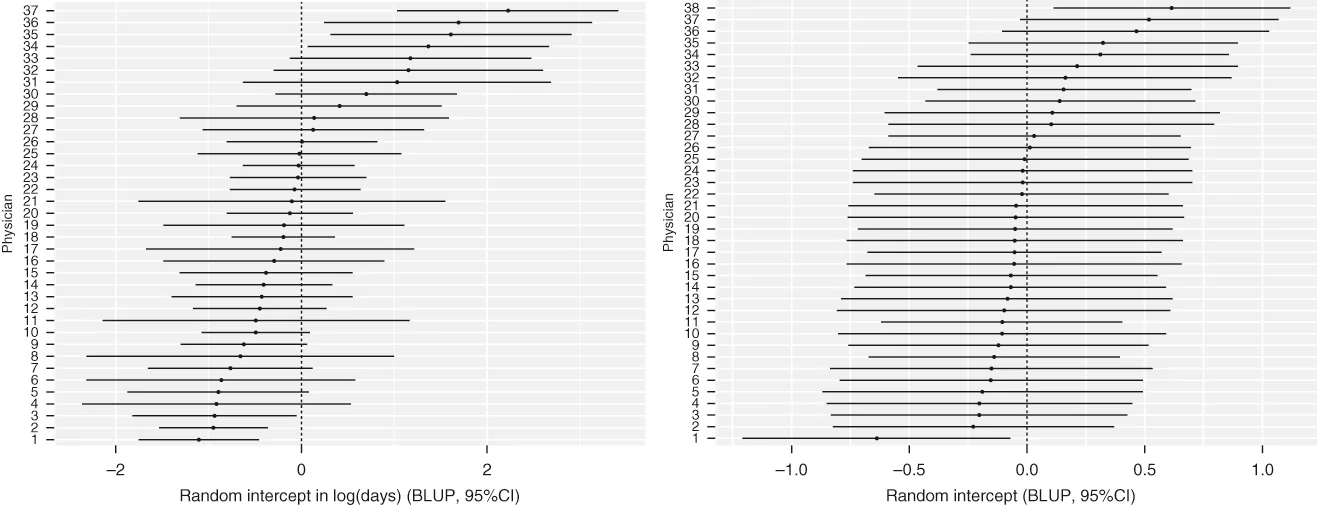
**Caterpillar plot of time to biopsy by physician and steroid prescribing by physician.** (Left Panel) Each horizontal line represents an individual physician. The dot indicates the physician-specific point estimate, and the line represents the 95% CI for the random intercept. Random effects were estimated as BLUPs from a multivariable mixed-effects linear regression model, with the natural logarithm of the time to kidney biopsy as the dependent variable and a random intercept specified for each physician. Estimates reflect between-physician variation in the time to kidney biopsy after adjustment for measured covariates. (Right Panel) Each horizontal line represents an individual physician. The dot indicates the physician-specific point estimate, and the line represents the 95% CI for the random intercept. Random effects were estimated as BLUPs from a multivariable mixed-effects logistic regression model, with glucocorticoid prescription as the dependent variable and a random intercept specified for each physician. Estimates reflect between-physician variation in the probability of glucocorticoid prescription after adjustment for measured covariates. BLUP, best linear unbiased predictor; CI, confidence interval.

The ICC from the final model was 0.31, indicating that 31% of the variability in time to kidney biopsy was attributable to between-physician variability in practice. The MTR from this model was 2.65, indicating that the time to biopsy for a given patient with the same characteristics could be 2.65-fold longer if seen by a slow-to-biopsy physician, versus a quick-to-biopsy physician. Factors associated with time to kidney biopsy included higher proteinuria, higher eGFR, and rural residence. Higher proteinuria was associated with a shorter time to biopsy; specifically, a doubling of proteinuria was associated with a 19% shorter time to biopsy (95% CI, 5% to 30%) in urban-dwelling patients. Higher eGFR was associated with a 52% longer time to biopsy (95% CI, 25% to 88%), and rural residence was associated with a 62% longer time to biopsy (95% CI, 9% to 139%). The model results are summarized in Table 3, and the random effects by physician are presented in Figure 2, Left Panel.

**Table 3:** Multivariable mixed-effects linear regression model for time to kidney biopsy in IgA nephropathy

Variable	Time Ratio	Percent Difference	95% CI	*P* Value
Intercept	68.6^a^	—	30.9 to 154.6^a^	<0.001^a^
eGFR	1.52^a^	52% longer^a^	1.25 to 1.88^a^	<0.001^a^
Proteinuria (log)	Urban: 0.76^a^	26% shorter^a^	0.59 to 0.92^a^	0.007^a^
	Rural: 0.48^a^	52% shorter^a^	0.26 to 0.86^a^	0.02^a^
Rural dwelling	1.62^a^	62% longer^a^	1.09 to 2.39^a^	0.02^a^
Age	1.22	22% longer	0.99 to 1.51	0.07
Sex	1.26	26% longer	0.87 to 1.80	0.23
Diabetes	1.34	34% longer	0.84 to 2.14	0.22
Hypertension	1.49	49% longer	0.89 to 2.51	0.13
MTR	2.65		1.99 to 3.79	
SD (intercept|physician)				1.02
SD (observations)				1.52
ICC		0.31	0.14 to 0.45	
Marginal R^2^				0.13
Conditional R^2^				0.40

Age and eGFR are mean centered; proteinuria is log transformed; outcome is log(days to biopsy).

Time ratios represent multiplicative differences in time to biopsy. Values >1 indicate longer time to biopsy and values <1 indicate shorter time. Percent differences are calculated as (time ratio−1)×100. Proteinuria was modeled on the log scale; estimates therefore reflect proportional changes (*e.g*., doubling of proteinuria). CI, confidence interval; ICC, intraclass correlation coefficient; MTR, median time ratio.

a Denotes statistically significant at alpha = 0.05.

### Association of Residence (Location) with Time to Biopsy

**Figure 3:**
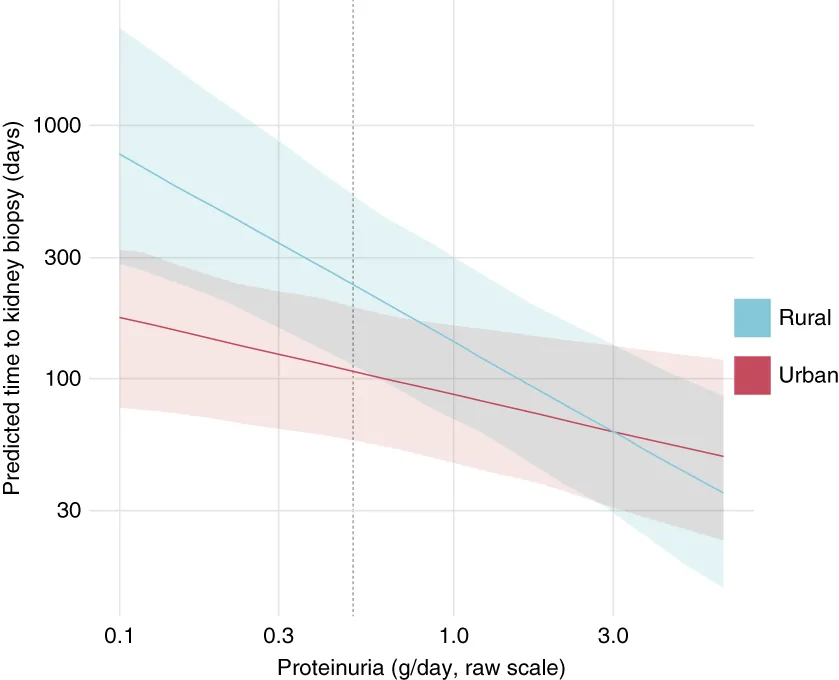
**Predicted time to kidney biopsy by proteinuria, stratified by location of residence.** Predicted values were generated from the fixed effects of the linear mixed-effects model, with continuous covariates (age and eGFR at biopsy) held at their population mean values (*i.e*., 0 after centering). Predictions represent population-average estimates for an average physician, defined as a nephrologist with a random intercept of 0 (the mean of the physician-level random-effects distribution). Shaded areas represent 95% prediction intervals. The dotted line represents proteinuria 0.5 g/d.

We examined the association between proteinuria, rural and urban residence, and time to biopsy. Figure 3 illustrates the modeled relationship between proteinuria and time to kidney biopsy, stratified by location of residence. The interaction between proteinuria and dwelling location was statistically significant, indicating that the effect of proteinuria on biopsy timing differed by residence. Specifically, for a proportional increase in proteinuria, the reduction in time to biopsy was 26% greater in rural compared with urban patients (95% CI, 7% to 56%). Although predicted time to biopsy decreased in both rural and urban patients, among those with subnephrotic proteinuria, rural patients were predicted to experience substantially longer time to biopsy. Among patients with 1 g/d of proteinuria, the estimated time to kidney biopsy for a typical patient was 86 days for those living in urban areas and 139 days for those in rural areas. For patients with 0.5 g/d of proteinuria, biopsy was predicted to occur substantially later, at 107 days for urban patients and 233 days for rural patients.

### Factors Associated with and Odds of Glucocorticoid Prescription

Overall, 23% of patients received glucocorticoids. Treated patients had higher proteinuria (2.41 g/d [IQR, 1.53–4.06] versus 1.60 g/d [IQR, 1.00–2.38]) and were biopsied sooner (34 days [IQR, 8–182] versus 85 days [IQR, 22–368]). The prevalence of Oxford T1/T2 lesions was similar between groups, whereas crescents were more common among treated patients (54% versus 18%). In a prespecified subset of patients meeting a proxy definition of KDIGO-recommended steroid eligibility (*n*=66), only three patients (4%) received glucocorticoids. Compared with the overall cohort, these patients had more advanced disease at baseline, with lower eGFR (median 35 versus 47 ml/min per 1.73 m^2^), higher proteinuria (2.15 versus 1.71 g/d), and a longer time to biopsy (median 116 versus 63 days). They also had a higher burden of chronic histologic changes, with 38% demonstrating >50% IFTA (Supplemental Table 7).

We fit three multivariable mixed-effects logistic regression models to assess the association between covariates and the likelihood of glucocorticoid prescription. The model including proteinuria and eGFR at biopsy, age, presence of diabetes and hypertension, presence of crescents, and Oxford T score had the lowest AIC and highest marginal and conditional R^2^ and was therefore considered the final model. The candidate models are summarized in Supplemental Table 6. The ICC of the final model was 0.07, indicating that 7% of the variability in glucocorticoid prescribing was attributable to between-physician variation in practice. The MOR was 1.59, indicating that the odds of glucocorticoid prescription for a given patient with identical clinical and pathologic characteristics could vary by as much as 59% if seen by a high-prescribing physician versus a low-prescribing physician. Factors associated with the likelihood of glucocorticoid prescription included proteinuria at biopsy (odds ratio [OR], 1.36; 95% CI, 1.09 to 1.63), eGFR (OR, 0.99; 95% CI, 0.98 to 1.00), crescents on biopsy (OR, 6.20; 95% CI, 2.12 to 10.28), and an Oxford T2 lesion (OR, 0.33; 95% CI, 0.02 to 0.64). The ICC, MOR, and coefficients of the models remained similar in the population restricted to physicians with five or more patients. The model results are summarized in Table 4, and the random effects by physician are presented in Figure 2, Right Panel.

**Table 4:** Multivariable mixed-effects logistic regression models for glucocorticoid prescription in IgA nephropathy

Variable	Full Model	Sensitivity Model
Intercept	0.092 (−0.07 to 0.25)	0.129 (−0.11 to 0.37)
Proteinuria at biopsy	1.36 (1.09 to 1.63)^a^	1.28 (1.01 to 1.56)^a^
eGFR at biopsy	0.99 (0.98 to 1.00)^a^	0.99 (0.97 to 1.00)^a^
Age at biopsy	1.02 (0.99 to 1.04)	1.02 (0.99 to 1.04)
Diabetes	1.11 (0.18 to 2.03)	0.89 (0.08 to 1.69)
Hypertension	1.10 (0.37 to 1.84)	1.09 (0.32 to 1.86)
Oxford C1/2	6.20 (2.12 to 10.28)^a^	6.44 (1.97 to 10.91)^a^
Oxford T1	0.64 (0.12 to 1.16)	0.57 (0.08 to 1.06)
Oxford T2	0.33 (0.02 to 0.64)^a^	0.35 (0.01 to 0.68)^a^
SD (intercept|physician)	0.48	0.53
MOR	1.59 (1.00 to 2.32)	1.65 (1.00 to 2.39)
ICC	0.07 (0.02 to 0.15)	0.08 (0.02 to 0.16)
Marginal R^2^	0.244	0.233
Conditional R^2^	0.295	0.295

Models are presented as odds ratio (95% confidence interval).

Sensitivity model includes only physicians with five or more patients with IgA nephropathy. ICC, intraclass correlation coefficient; MOR, median odds ratio.

a Denotes statistically significant at alpha = 0.05.

## Discussion

In this population-based North American cohort of patients with IgAN, we found that a substantial proportion of the variability in both time to biopsy and likelihood of glucocorticoid prescription were attributable to the physician treating the patient with >2.6-fold variability in time to biopsy between physicians. Higher proteinuria was associated with shorter time to biopsy, and we observed an interaction with rural residence, where lower levels of proteinuria were associated with a significant increase in time to kidney biopsy. Higher proteinuria and the presence of crescents on kidney biopsy were associated with a higher likelihood of glucocorticoid prescription, while the presence of more than 50% IFTA was associated with a lower likelihood of glucocorticoid prescription.

To our knowledge, this is the first study in glomerular diseases to quantify between-physician variability in practice using mixed-effects models. Previous survey-based studies have suggested that physician adherence to guideline-recommended management of glomerular diseases is limited,^30,31^ but survey data are prone to response and recall bias^32^ and may not account for differences in case mix—physicians may care for patients with varying disease severity, making comparisons solely on the basis of self-reported treatment proportions misleading. By leveraging a population-based registry, we were able to examine factors associated with the timing of biopsy and the use of glucocorticoids, while directly quantifying the magnitude of variation between physicians, without the limitations inherent to survey-based research.

We quantified between-physician variability using two complementary approaches: the ICC and ratio-based measures to facilitate interpretation of the magnitude of variation. The physician-level ICC for time to biopsy was 0.31, indicating that nearly one third of the total variability in biopsy timing was attributable to differences between physicians, comparable with physician-level variation reported for CKD quality indicators in primary care (ICC, 0.24–0.31)^33^ and substantially higher than clustering observed for dialysis initiation across facilities (ICC≈0.03 after adjustment).^34^ However, the marginal and conditional R^2^ values (0.18 and 0.40) suggest substantial unexplained variability. The marked right skewness of time to biopsy further indicates heterogeneity in disease presentation and referral patterns not captured by measured covariates. Accordingly, these estimates should be interpreted with caution and may reflect historical uncertainty regarding optimal biopsy timing in IgAN.

For glucocorticoid prescription, the ICC was 0.07, suggesting a smaller but still meaningful degree of between-physician variability, comparable with a study of variability in antibiotic prescribing between Australian primary care practices.^35^ Although differences in case mix may contribute to the observed variation, patients at our center are allocated to physicians on a rotating basis, which likely reduces systematic differences in case mix. Nevertheless, residual variation in patient severity may still account for some of the observed variability.

We also expressed physician-level variability using the MOR and analogous MTR for interpretability. The MOR for glucocorticoid prescription was 1.59, similar to the values reported in the previously mentioned Australian antibiotic prescription study (MOR, 1.32–1.53).^35^ For time to biopsy, the MTR was 2.65, indicating that, for two otherwise similar patients, the time to biopsy could differ by a factor of 2.65 solely on the basis of the physician they saw, a striking degree of variation, although with no direct precedent in the existing literature.

We identified an interaction between rurality and the association of proteinuria with time to kidney biopsy. In Manitoba, kidney biopsy services are centralized in Winnipeg; rural and northern patients face additional travel and coordination steps (even with the Northern Patient Transportation Program^36^), alongside documented access and cultural safety barriers.^37^ These factors likely magnify delays when proteinuria is modest (and where biopsy decisions are perceived to be more discretionary), while the gap narrows at higher proteinuria when clinical urgency overrides inertia because of logistical challenges.

These findings have important knowledge translation implications in the context of the 2025 KDIGO guidelines, which emphasize earlier biopsy and increasing use of disease-modifying therapies across the disease spectrum. The practice patterns observed in this study predate these major shifts in the evidence base, including recognition of long-term risk even at lower levels of proteinuria and the demonstration of treatment effects of immunosuppressive therapies across a broad range of histopathologic findings. The observed delays in biopsy among patients with lower proteinuria suggest that underestimation of lifetime kidney risk may have contributed to deferred diagnostic evaluation. Our findings reinforce how evolving understanding of risk and treatment efficacy must be translated not only into prescribing decisions but also into upstream diagnostic behaviors. When few options for treatment were available, obtaining a specific diagnosis may be seen as unnecessary. With recent advancements in IgAN risk stratification and therapeutics, it has become indispensable.

We also found that pathology features, in particular active crescentic glomerular injury on kidney biopsy, were strong predictors of the likelihood of prescription of glucocorticoids. Notably, recent trials have not demonstrated effect modification by histopathologic features,^13,14,38^ suggesting that treatment benefit is not confined to specific biopsy findings. While, in the absence of such evidence, it was logical for physicians to base treatment decisions on pathologic severity, this rationale is less tenable in the context of contemporary data showing benefit across a broader spectrum of disease. Together, these developments underscore the evolving landscape of IgAN management and the need for deliberate, evidence-informed knowledge translation strategies to align clinical practice with current understanding of disease risk and treatment efficacy.

The low rate of glucocorticoid prescribing among patients meeting proxy KDIGO eligibility criteria warrants careful interpretation. On one hand, this may reflect therapeutic inertia or hesitancy to escalate beyond supportive care, particularly given the known toxicity profile of prolonged high-dose glucocorticoids. On the other hand, patients in this subgroup had more advanced disease at biopsy, including substantially reduced kidney function and a higher prevalence of chronic histologic injury, which may have influenced clinicians toward a perception of limited treatment benefit or futility. In addition, our proxy eligibility definition may have captured patients in whom glucocorticoids would not have been clinically appropriate despite meeting guideline-based criteria.

This study has strengths, including its population-based design, broad inclusion of nephrologists, and use of multilevel models to disentangle patient- and physician-level contributions to care variation. Linkage of administrative and pathology data allowed comprehensive adjustment for clinical and histologic features. Limitations include the single-center setting; incomplete preindex laboratory data preventing modeling of prior eGFR and proteinuria trajectories; and the absence of ethnicity,^39,40^ hematuria, and other patient-level contextual factors that may influence decision making. Not all Oxford components were available, limiting our ability to assess associations with M and E lesions. Physician characteristics, such as training background or experience, could not be evaluated because of anonymization. In addition, the observed variation in practice patterns may reflect unmeasured influences, including patient preferences, differences in referral patterns, nonmedical factors, temporal changes in individual physician practice, and residual confounding. Finally, because our data source was restricted to patients captured within the biopsy registry, we did not have access to the broader population of patients evaluated in nephrology clinics in whom IgAN was suspected but a biopsy was not performed. As such, our analysis is conditional on having undergone biopsy and we were unable to assess variability in the decision of whether to biopsy or the time to biopsy among all potentially eligible patients at the point of initial clinical presentation.

In this population-based study of patients with IgAN, we identified substantial variability between physicians in both timing of kidney biopsy and use of glucocorticoids. Major efforts in physician education will be needed to ensure that timely biopsy and treatment initiation occur in the new era of multiple effective therapies for IgAN and new clinical practice guidelines.

## Supplementary Material

## Data Availability

Original data generated for the study are or will be made available in a repository subject to controlled access. Data Type: Health Care Data. Repository Name: MCHP Population Data Repository. Reason for Restriction: Data are available from the MCHP, University of Manitoba for researchers who meet the criteria for access to confidential data. All data used in our analysis are publicly available and are held by the Government of Manitoba or contracted agencies (*e.g*., laboratory services). Access to these data requires approval by the Committee for Harmonized Health Impact, Privacy, and Ethics Review, the Government of Manitoba’s Provincial Health Research Privacy Committee, and Shared Health Services of Manitoba. Legal restrictions prevent the use of these data without approval from each of the individual trustees. Specific datasets used in this project include: Shared Health Diagnostic Services of Manitoba (laboratory tests); Hospital Discharge Abstracts (hospitalizations); Manitoba Health Insurance Registry (registration coverage periods, demographics); Manitoba Health Medical Services (physician claims/billings); Drug Program Information Network (pharmacy dispensing); Manitoba GN Registry (biopsy results). All of these data are provided in de-identifiable format and linked through a remote access server maintained by the MCHP (https://umanitoba.ca/manitoba-centre-for-health-policy/). Email address: mchp.access@umanitoba.ca.

## Supplemental Material

Supplemental Table 1. Data collected in the Manitoba Glomerular Diseases Registry.

Supplemental Table 2. List of study databases.

Supplemental Table 3. Definitions of nephrology visit, dialysis, kidney transplantation, and comorbidities.

Supplemental Table 4. Characteristics of patients by location of initial nephrology visit.

Supplemental Table 5. Candidate multivariable mixed-effects linear regression models for time to kidney biopsy in IgAN.

Supplemental Table 6. Candidate multivariable mixed-effects logistic regression models for steroid prescription in IgAN.

Supplemental Table 7. Characteristics of KDIGO cohort compared with the overall cohort.
