# Supplementary material for: Physician Variability in Time to Kidney Biopsy and Glucocorticoid Prescription in IgA Nephropathy: A Population-Based Cohort Study
Source: Kidney360. 2026 May 14;7(8):1854–63. doi: 10.34067/KID.0000001232 (PMC13567853; doi:10.34067/KID.0000001232)
Supplement: Supplementary file 1 [file kidney360-7-1854-s001.pdf]

ASN Journal Disclosure Form

As per ASN journal policy, I have disclosed any financial relationships or commitments I have held in the past 36 months as included below. I have listed my Current Employer below to indicate there is a relationship requiring disclosure. If no relationship exists, my Current Employer is not listed.

B. Barr reports the following:
Employer: University of Manitoba; Consultancy: Otsuka Canada; Novartis; GSK; SOBI; Roche; and Research Funding: Otsuka.

I understand that the information above will be published within the journal article, if accepted, and that failure to comply and/or to accurately and completely report the potential financial conflicts of interest could lead to the following: 1) Prior to publication, article rejection, or 2) Post-publication, sanctions ranging from, but not limited to, issuing a correction, reporting the inaccurate information to the authors' institution, banning authors from submitting work to ASN journals for varying lengths of time, and/or retraction of the published work.

Name: Bryce Barr
Manuscript ID: K360-2026-000190R1
Manuscript Title: Physician variability in time to kidney biopsy and glucocorticoid prescription in IgA nephropathy: a population-based cohort study
Date of Completion: April 2, 2026
Disclosure Updated Date: April 2, 2026

ASN
American Society of Nephrology
JASN
Journal of the American Society of Nephrology
KIDNEY360
Accessing Our World From Every Angle
CJASN
Clinical Journal of the American Society of Nephrology

ASN Journal Disclosure Form

As per ASN journal policy, I have disclosed any financial relationships or commitments I have held in the past 36 months as included below. I have listed my Current Employer below to indicate there is a relationship requiring disclosure. If no relationship exists, my Current Employer is not listed.

I. Gibson reports the following:
Employer: University of Manitoba; and Consultancy: argenx.

I understand that the information above will be published within the journal article, if accepted, and that failure to comply and/or to accurately and completely report the potential financial conflicts of interest could lead to the following: 1) Prior to publication, article rejection, or 2) Post-publication, sanctions ranging from, but not limited to, issuing a correction, reporting the inaccurate information to the authors' institution, banning authors from submitting work to ASN journals for varying lengths of time, and/or retraction of the published work.

Name: Ian W. Gibson
Manuscript ID: K360-2026-000190R1
Manuscript Title: Physician variability in time to kidney biopsy and glucocorticoid prescription in IgA nephropathy: a population-based cohort study
Date of Completion: April 2, 2026
Disclosure Updated Date: March 12, 2026

ASN American Society of Nephrology JASN Journal of the American Society of Nephrology KIDNEY360 Accessing Our World From Every Angle CJASN Clinical Journal of the American Society of Nephrology

ASN Journal Disclosure Form

As per ASN journal policy, I have disclosed any financial relationships or commitments I have held in the past 36 months as included below. I have listed my Current Employer below to indicate there is a relationship requiring disclosure. If no relationship exists, my Current Employer is not listed.

H. Hildebrand reports the following:
Employer: University of Manitoba

I understand that the information above will be published within the journal article, if accepted, and that failure to comply and/or to accurately and completely report the potential financial conflicts of interest could lead to the following: 1) Prior to publication, article rejection, or 2) Post-publication, sanctions ranging from, but not limited to, issuing a correction, reporting the inaccurate information to the authors' institution, banning authors from submitting work to ASN journals for varying lengths of time, and/or retraction of the published work.

Name: Hailey Victoria Hildebrand
Manuscript ID: K360-2026-000190R1
Manuscript Title: Physician variability in time to kidney biopsy and glucocorticoid prescription in IgA nephropathy: a population-based cohort study
Date of Completion: April 2, 2026
Disclosure Updated Date: April 2, 2026

ASN American Society of Nephrology | JASN Journal of the American Society of Nephrology | KIDNEY360 Accessing Our World From Every Angle | CJASN Clinical Journal of the American Society of Nephrology

ASN Journal Disclosure Form

As per ASN journal policy, I have disclosed any financial relationships or commitments I have held in the past 36 months as included below. I have listed my Current Employer below to indicate there is a relationship requiring disclosure. If no relationship exists, my Current Employer is not listed.

N. Tangri reports the following:
Employer: University of Manitoba; Consultancy: AstraZeneca, Boehringer Ingelheim, Otsuka Pharmaceutical Co, Ltd, Bayer, ProKidney, Roche, Vera, American Society of Nephrology; Ownership Interest: PulseData Inc, Mesentech Inc., Klinrisk, Quanta, ProKidney; Research Funding: Bayer, AstraZeneca, Boehringer Ingelheim, Otsuka Pharmaceutical Co, Ltd; Patents or Royalties: Co-investor- A microfluidic device for point of care detection of urine albumin. ; Pending patent- Use of lab data to predict CKD progression.;; and Advisory or Leadership Role: National Kidney Foundation;.

I understand that the information above will be published within the journal article, if accepted, and that failure to comply and/or to accurately and completely report the potential financial conflicts of interest could lead to the following: 1) Prior to publication, article rejection, or 2) Post-publication, sanctions ranging from, but not limited to, issuing a correction, reporting the inaccurate information to the authors' institution, banning authors from submitting work to ASN journals for varying lengths of time, and/or retraction of the published work.

Name: Navdeep Tangri
Manuscript ID: K360-2026-000190R1
Manuscript Title: Physician variability in time to kidney biopsy and glucocorticoid prescription in IgA nephropathy: a population-based cohort study
Date of Completion: April 2, 2026
Disclosure Updated Date: April 2, 2026
