# Supplementary material for: Physician Variability in Time to Kidney Biopsy and Glucocorticoid Prescription in IgA Nephropathy: A Population-Based Cohort Study
Source: Kidney360. 2026 May 14;7(8):1854–63. doi: 10.34067/KID.0000001232 (PMC13567853; doi:10.34067/KID.0000001232)
Supplement: Supplementary file 2 [file kidney360-7-1854-s002.pdf]

Supplemental Table 1. Data collected in the Manitoba Glomerular Diseases Registry

Data	Type	Possible values / Usual range of values
Personal Health Identification Number	numeric	9-digit number
Last name	text	1 or 2 names
First name	text	1 or 2 names
Date of birth	numeric	DD/MM/YYYY
Age	numeric	Up to 3-digit number
Date biopsy collected / resultreceived	numeric	DD/MM/YYYY
Diagnosis	text	variable
Cores	numeric	1-5
Total glomeruli	numeric	0-50
Sclerosed glomeruli	numeric	0-50
Glomeruli with segmental sclerosis	numeric	0-50
Glomeruli with fibrinoid necrosis	numeric	0-50
Glomeruli with cellular crescents	numeric	0-50
Glomeruli with fibrocellular crescents	numeric	0-50
Glomeruli with fibrous crescents	numeric	0-50
IFTA (interstitial fibrosis and tubular atrophy)	numeric or text	0%-100% or [mild, patchy, moderate, diffuse, significant]
Intimal fibrosis	text	no, yes, mild, moderate, severe
Vasculitis	binary	no, yes
Immunofluorescence	binary	no, yes
IgG	text	trace, 1+. 2+. 3+, +/-
IgA	text	trace, 1+. 2+. 3+, +/-
IgM	text	trace, 1+. 2+. 3+, +/-
AHIg	text	trace, 1+. 2+. 3+, +/-
C3	text	trace, 1+. 2+. 3+, +/-
C1q	text	trace, 1+. 2+. 3+, +/-
Fibrinogen	text	trace, 1+. 2+. 3+, +/-
Kappa	text	trace, 1+. 2+. 3+, +/-
Lambda	text	trace, 1+. 2+. 3+, +/-
Foot Process Effacement (FPE)	text	no, yes, mild, moderate, focal, extensive, diffuse, widespread, severe
Subepithelial deposits	binary	no, yes
Subendothelial deposits	binary	no, yes
Intramembranous deposits	binary	no, yes
Mesangial deposits	binary	no, yes
PLA2R	binary	negative, positive
Oxford M/E/S/T/C	numeric	0, 1, 2
Lupus class	roman num.	I, II, III, IV, V
Lupus AI (activity index)	numeric	0-24
Lupus CI (chronicity index)	numeric	0-12

Supplemental Table 2. List of study databases

Database	Study Years	Data Provider	Data Fields/Variables	Description
Shared Health Diagnostic Services	January 1, 2002 to December 31, 2023	Diagnostic Services, Shared Health	• Scrambled PHIN<br>• Lab Results – Chemistry/Hematology<br>• Date of Collection<br>• Test Description<br>• Test Numeric Results	Laboratory tests and results
Hospital Discharge Abstracts	January 1, 2002 to December 31, 2023	Manitoba Health, Seniors and Active Living	• Scrambled PHIN<br>• Admission Date<br>• Separation Date<br>• Diagnosis Codes<br>• Intervention Codes<br>• Intervention Dates<br>• Length of Stay	Hospital discharge data
Manitoba Health Insurance Registry	January 1, 2002 to December 31, 2023	Manitoba Health, Seniors and Active Living	• Scrambled PHIN<br>• Sex<br>• Date of Birth<br>• Coverage Dates (start/end)<br>• Reason for Coverage Cancellation<br>• Postal Code<br>• Municipality Code	Patient registry and provincial health insurance coverage dates
Medical Health Services Claims	January 1, 2002 to December 31, 2023	Manitoba Health, Seniors and Active Living	• Scrambled PHIN<br>• Date of Service<br>• Tariff Code<br>• Diagnosis Code<br>• Specialty Sub-bloc<br>• Number of Services Provided<br>• Physician type / specialty	Physician claims data
Drug Program Information Network	January 1, 2002 to December 31, 2023	Manitoba Health, Seniors and Active Living	• Scrambled PHIN<br>• Date Prescription Provided<br>• Days Supplied<br>• DIN	Drug prescriptions

			• Dosage (Strength)<br>• ATC Code<br>• Drug Cost Claimed<br>• Drug Cost Paid<br>• Product Description<br>• Product Name	
Manitoba Glomerular Diseases Registry	January 1, 2002 to December 31, 2021	Diagnostic Services, Shared Health	• See Supplemental Table 1	Information on all incident GN biopsies

Supplemental Table 3. Definitions of nephrology visit, dialysis, kidney transplantation, and comorbidities

Variable	Medical Health Services Claims Tariff Codes	Hospital Discharge Abstracts Diagnosis Codes
Nephrology Visit	The first code from the following list:<br>8595, 8550, 8540<br>arising from bloc 016	--
Hypertension	1 hospital diagnosis or 2 outpatient claims within 2 years	
		ICD-9-CM: 401, 402, 403, 404, 405<br>ICD-10-CA: I10, I11, I12, I13, I15
Diabetes	1 hospital diagnosis or 2 outpatient claims within 2 years	
		ICD-9-CM: 250<br>ICD-10-CA: E10, E11, E13, E14

Supplemental Table 4. Characteristics of patients by location of initial nephrology visit

Characteristic	Inpatient visit (n = 70)	Outpatient visit (n = 310)
**Age**	40 (29, 60)	42 (31, 53)
**Female sex**	25 (36%)	131 (42%)
**eGFR (mL/min/1.73 m^2)**	20 (9, 75)	59 (35, 90)
**Proteinuria (g/day)**	3.84 (1.29, 5.40)	1.62 (0.91, 2.63)
**Rural dwelling**	37 (53%)	114 (37%)
**SES quintile**		
**1**	34 (45%)	101 (33%)
**2**	20 (26%)	64 (21%)
**3**	10 (15%)	55 (18%)
**4**	8 (12%)	57 (19%)
**5**	2 (3%)	31 (10%)
**Diabetes**	23 (33%)	63 (20%)
**Hypertension**	53 (76%)	267 (86%)
**Days to biopsy (mean, SD)**	371 (930)	534 (1,151)
**Days to biopsy (median, IQR)**	10 (6, 152)	86 (29, 368)

Supplemental Table 5. Candidate multivariable mixed-effects linear regression models for time to kidney biopsy in IgA nephropathy.

	Empty Model	Model A	Model B	Model C	Model D	Final Model
**Intercept**	4.717 (4.30, 5.13)	4.94 (4.47, 5.41)	4.902 (4.41, 5.39)	4.28 (3.64, 4.92)	**4.45 (3.57, 5.33)**	**4.23 (3.43, 5.04)**
**Age**		0.18 (-0.02, 0.37)	0.18 (-0.02, 0.38)	0.08 (-0.13, 0.28)	0.14 (-0.06, 0.34)	0.20 (-0.01, 0.41)
**eGFR**		0.11 (-0.09, 0.31)	0.11 (-0.09, 0.31))	0.12 (-0.08, 0.32)	0.22 (0.04, 0.40)	**0.42 (0.22, 0.63)**
**Proteinuria**		**-0.27 (-0.50, -0.04)**	**-0.28 (-0.50, -0.05)**	**-0.31 (-0.54, -0.08)**	**-0.41 (-0.63, -0.19)**	**-0.30 (-0.52, -0.08)**
**Proteinuria (Rural Dwelling)**					**-0.65 (−1.05, −0.25)**	**-0.74 (-1.34, -0.15)**
**Rural dwelling**			0.10 (-0.28, 0.48)	0.10 (-0.28, 0.48)	**0.58 (0.21, 0.95)**	**0.48 (0.09, 0.87)**
**Female sex**				0.21 (-0.15, 0.61)	0.19 (-0.16, 0.54)	0.23 (-0.14, 0.59)
**Diabetes**				0.29 (-0.22, 0.81)	0.08 (-0.33, 0.49)	0.29 (-0.17, 0.76)
**Hypertension**				**0.70 (0.18, 1.21)**	0.41 (-0.10, 0.92)	0.40 (-0.12, 0.92)
**Era**						
**2002-2009**					Ref	
**2010-2015**					-0.19 (-0.53, 0.32	
**2016-2021**					-0.12 (-0.57, 0.33)	
**SD(Intercept|Physician)**	1.05	1.17	1.18	1.11	0.93	1.02
**SD(Observations)**	1.80	1.77	1.77	1.76	1.50	1.52
**Median Time Ratio**	2.72	3.01	3.02	2.84	2.43	2.65
**ICC**	0.25	0.31	0.31	0.29	0.28	0.31
**AIC**	1574	1578	1581	1578	1349	1228

**Marginal R^2**	0	0.022	0.022	0.038	0.11	0.13
**Conditional R^2**	0.25	0.32	0.32	0.32	0.36	0.40

Age and eGFR are mean-centred; proteinuria is log-transformed; outcome is log(days)
eGFR, estimated glomerular filtration rate; SD, standard deviation; AIC, Akaike information criterion; intraclass correlation coefficient

Supplemental Table 6. Candidate multivariable mixed-effects logistic regression models for steroid prescription in IgA nephropathy

	Empty Model	Model A	Model B	Model C
**Intercept**	**0.26 (0.17, 0.35)**	**0.09 (-0.04, 0.21)**	**0.06 (-0.04, 0.16)**	0.092 (-0.07, 0.25)
**Proteinuria at biopsy**		**1.42 (1.16, 1.68)**	**1.44 (1.17, 1.72)**	**1.36 (1.09, 1.63)**
**eGFR at biopsy**		1.00 (0.99, 1.01)	1.00 (0.99, 1.01)	**0.99 (0.98, 1.00)**
**Age at biopsy**		1.01 (0.99, 1.03)	1.01 (0.99, 1.03)	1.02 (0.99, 1.04)
**Diabetes**			0.74 (0.18, 1.29)	1.11 (0.18, 2.03)
**Hypertension**			1.30 (0.11, 2.50)	1.10 (0.37, 1.84)
**Oxford C1/2**				**6.20 (2.12, 10.28)**
**Oxford T1**				0.64 (0.12, 1.16)
**Oxford T2**				**0.33 (0.02, 0.64)**
**SD(Intercept|Physician)**	0.41	0.43	0.45	0.48
**Median Odds Ratio**	1.47	1.51	1.54	1.59
**AIC**	337	321	327	310
**Marginal R^2**	0	0.08	0.09	0.24
**Conditional R^2**	0.05	0.13	0.15	0.30
**ICC**	0.036	0.052	0.055	0.068

Models are presented as odds ratio (95% confidence interval)
eGFR, estimated glomerular filtration rate; SD, standard deviation; AIC, Akaike information criterion; ICC, intraclass correlation coefficient

Supplemental Table 7. Characteristics of KDIGO cohort compared to overall cohort

Characteristic	Overall (n = 380)	KDIGO cohort (n = 66)
**Age at biopsy**	42 (30, 54)	43 (28, 52)
**Female sex**	156 (41%)	32 (48%)
**eGFR at initial visit (mL/min/1.73 m^2)**	52 (29, 82)	45 (17, 79)
**eGFR at biopsy (mL/min/1.73 m^2)**	47 (25, 78)	35 (13, 64)
**Proteinuria at initial visit (g/day)**	1.88 (1.21, 3.31)	2.41 (1.74, 4.65)
**Proteinuria at biopsy (g/day)**	1.71 (1.05, 2.71)	2.15 (1.56, 4.09)
**Days to kidney biopsy**	63 (20, 321)	116 (16, 547)
**Diabetes**	64 (17%)	9 (14%)
**Hypertension**	210 (55%)	42 (64%)
**Rural dwelling**	148 (39%)	37 (56%)
**IFTA**		
**0-25%**	175 (46%)	20 (30%)
**26-50%**	90 (24%)	21 (32%)
**>50%**	115 (30%)	25 (38%)
**Crescents present**	100 (26%)	26 (39%)

Continuous variables are expressed as median (interquartile range)
Categorical variables are expressed as percentages
eGFR, estimated glomerular filtration rate
IFTA, interstitial fibrosis and tubular atrophy
KDIGO, Kidney Diseases: Improving Global Outcomes

Supplemental methods: Calculating intraclass correlation coefficient, median time ratio and median odds ratio

The ICC represents the proportion of the total variance in the (log-transformed) outcome attributable to between-physician differences[35]:

$$\mathrm{ICC} = \frac{\sigma_u^2}{\sigma_u^2 + \sigma_e^2}$$

Where σ_u^2 represents the variance of the random intercept (between-physician variability) and σ_e^2 represents the residual variance.

The Median Odds Ratio and Median Time Ratio are calculated in the same way[29]. Under the assumption that random intercepts are normally distributed:

1. The difference between two physicians' random intercepts follows

$$\Delta u = u_i - u_j \sim N(0,\, 2\sigma_u^2)$$

2. The median absolute difference between two physicians' intercepts on the log-scale is

$$\mathrm{Median}(|\Delta u|) = \sqrt{2\sigma_u^2} \times \Phi^{-1}(0.75)$$

   where $\Phi^{-1}(0.75) = 0.6745$ is the 75th percentile of the standard normal distribution.

3. Exponentiating yields the MTR (or MOR in the case of the logistic model):

$$\mathrm{MTR} = \exp\left(\sqrt{2\sigma_u^2} \times 0.6745\right)$$

In the logistic model, the ICC is defined as[28]:

$$\mathrm{ICC} = \frac{\tau^2}{\tau^2 + \pi^2/3}$$

Where τ^2 represents the variance of the physician-level random intercept and $\pi^3/3$ represents the residual variance on the logistic (logit) scale, corresponding to the variance of the standard logistic distribution (~3.29).

Supplemental methods: Creation of the KDIGO cohort

To be included in the KDIGO cohort, patients were required to meet two eligibility criteria for inclusion: 1) Persistent proteinuria >1 g/day documented between 180 days prior to biopsy and 270 days after biopsy, satisfied either by (a) two values ≥1 g/day separated by ≥30 and ≤180 days, **or** (b) a time-weighted average proteinuria >1 g/day sustained over ≥90 days. The first qualifying proteinuria value was carried forward as a covariate. 2) RAAS blockade criterion: Proportion of days covered (PDC) of at least 80% with ACEi/ARB over the 90 days preceding the eligibility date. The eligibility index date was defined as the later of the second qualifying proteinuria value or the biopsy date, whichever was soonest. The cohort characteristics are summarized in Supplemental Table 7.

We also explored the timing of glucocorticoid prescription and identified 15 patients who commenced glucocorticoids in the 60 days prior to the kidney biopsy. Only 4 of these continued glucocorticoids after the kidney biopsy. As the study design precludes knowledge regarding the circumstances of glucocorticoid prescription, these 4 patients were excluded from the primary analysis, and a sensitivity analysis was performed where they were included and considered as treated with glucocorticoids, with no substantial change in effect estimates.
